# Laparoscopic and Open Nephron-Sparing Surgery for Radius Exophytic/Endophytic Nearness Anterior/Posterior Location Nephrometry Score 7 and Higher Kidney Tumors: A Comparison of Oncological and Functional Outcomes Using the Pentafecta Score

**DOI:** 10.5152/tud.2023.22233

**Published:** 2023-05-01

**Authors:** Carlo Giulioni, Daniele Castellani, Manuel Di Biase, Vincenzo Ferrara, Andrea Benedetto Galosi

**Affiliations:** 1 Department of Urology, Azienda Ospedaliera Universitaria Delle Marche,Polytecnic University of Marche, Ancona, Italy; 2 Department of Urology, “Carlo Urbani” Hospital, Jesi, Italy

**Keywords:** Renal tumor, nephron-sparing surgery, open surgery, laparoscopy, clampless, Pentafecta score

## Abstract

**Objective:**

This study aimed to evaluate oncological and functional outcomes of nephron-sparing surgery by comparing open and laparoscopic approaches in a consecutive series of patients with intermediate and high complexity renal masses.

**Materials and Methods:**

We retrospectively reviewed all nephron-sparing surgery cases in 2 referral centers from January 2013 to January 2020. Tumor complexity was graded according to radius exophytic/endophytic nearness anterior/posterior location nephrometry score. Patients with a single kidney tumor with a radius exophytic/endophytic nearness anterior/posterior location score ≥ 7 were evaluated. Exclusion criteria were solitary kidney, multiple/bilateral tumors, and a low radius exophytic/endophytic nearness Anterior/Posterior location score (<7). Patients were divided according to the surgical approach: the laparoscopic tumor enucleation and the open wedge resection groups. The Trifecta and Pentafecta score achievement rates were assessed.

**Results:**

Two hundred thirteen patients were included in the analysis, 76 in laparoscopic tumor enucleation group and 137 in the open wedge resection group. There were no statistically significant differences in preoperative data between laparoscopic tumor enucleation and open wedge resection groups, except for the higher percentage of T1a masses in the latter group. The mean 24-hour blood loss and length of stay were higher in the open wedge resection group. Minor and major postoperative complication rates were comparable. No significant difference in terms of the Trifecta score was reported. Pentafecta score was achieved in 35/76 (46.1%) and 61/137 (44.5%) cases in the laparoscopic tumor enucleation and open wedge resection groups, respectively.

**Conclusion:**

Our study showed that laparoscopic tumor enucleation was associated with significantly lower blood and length of stay. Postoperative complications and the achievement of the Pentafecta score were similar in both surgical approaches.

**Main Points**

- Clampless nephron-sparing surgery for highly complex renal tumors is feasible in almost all cases both in open and laparoscopic approaches and does not lead to a high perioperative bleeding complication rate.
- Laparoscopic and open nephron-sparing surgery seems to guarantee similar oncological radicality for complex renal masses.
- A meticulous suturing of renal parenchyma is associated with a lower postoperative blood transfusion and trans-arterial embolization rate.

## Introduction

According to the current European Association of Urology guidelines, nephron-sparing surgery (NSS) is the standard treatment for kidney tumors up to 4 cm (T1a), both for oncological outcome and preservation of renal function.^1^ Nephron-sparing surgery should also be favored in tumors up to 7 (T1b) if resection is deemed technically feasible. Nowadays, open, laparoscopic, and robot-assisted approaches are considered acceptable, depending on the surgeon’s expertise and technological availability.^1^ Conversely, the complexity of partial nephrectomy is not based merely on the largest diameter of the tumor but also on renal tumor depth and its contiguity/infiltration to the collecting system. In the last decades, several scores have been popularized to predict the likelihood of operative complexity based on postoperative complications^2^ or warm ischemic time (WIT).^3^ Radius exophytic/endophytic nearness anterior/posterior location (RENAL) nephrometry score is one of the most used score.^4^ T1 tumors may vary in complexity, and the inexperienced surgeon may hesitate to perform NSS in more challenging cases.

The standard approach to NSS commonly requires main renal artery clamping to decrease intraoperative bleeding. However, renal clamping is associated with hypoxia^5^ and reperfusion damage,^6^ leading to kidney function deterioration. Clamping time should be as low as possible since more than 25 minutes of WIT was associated with 5% increased risks of acute renal failure and 6% risk of IV-stage chronic kidney disease (CKD) for each additional minute.^7^ Over the years, a progressive reduction in hypoxia has been applied through the increase in expertise and the introduction of super-selective clamping or clampless techniques that have shown to achieve similar perioperative safety with superior short-term renal function preservation compared to main artery clamping.^8^

Recently, a meta-analysis compared laparoscopic and open NSS, showing a less increased postoperative serum creatinine in the former, although a higher positive surgical margin (PSM) rate was reported.^9^ However, long-term renal function was not fully assessed.

Therefore, we aimed to assess medium-term functional and oncological outcomes comparing open and laparoscopic NSS in patients with a RENAL score tumor equal or higher than 7.

## Materials and Methods

### Patients

We retrospectively reviewed 2 collected databases of consecutive patients undergoing NSS in 2 tertiary referral centers (“Azienda Ospedaliero-Universitaria delle Marche,” Italy, and “Ospedale Carlo Urbani,” Italy) between January 2013 and January 2020. Inclusion criteria were adult patients with a single tumor with a RENAL score ≥ 7. Patients with incomplete data, a solitary kidney, low RENAL score (<7), and multiple/bilateral tumors were excluded. All patients were evaluated preoperatively by contrast-enhanced computed tomography scan or magnetic resonance imaging of the abdomen in case of contrast allergy or renal failure. Formal ethics committee approval was deemed unnecessary for this type of study in our institute because retrospective data collection was obtained for clinical purposes, and all the procedures were performed as part of routine care. The study was conducted following the 1964 Helsinki declaration and its later amendments. All patients signed an informed consent to gather their anonymized data.

### Collected Data

Radius exophytic/endophytic nearness anterior/posterior location nephrometric score was calculated for each patient. Comorbidities were assessed using the Charlson comorbidity index and the American Society of Anaesthesiologists (ASA) physical status classification system score. We gathered for all patients preoperative (demographics, comorbidity, ASA score, renal function, tumor side and dimension, nephrometry score), intraoperative (surgical time, WIT, rate of conversion to nephrectomy, complications), and postoperative (24-hour hemoglobin decrease, PSMs, complications, renal function) data. Patients were divided into 2 groups according to the surgical approach namely laparoscopic tumor enucleation (LTE) and open wedge resection (OWR) group.

Two experienced urologists (1 in each center) performed all the procedures, the first surgeon using the open retroperitoneal approach through the traditional flank incision between X and XI ribs and the second one the retroperitoneal laparoscopic approach as fully described previously.^10^ Tumor enucleation was attempted in all cases to preserve renal parenchyma as much as possible. The laparoscopic surgeon applied the retroperitoneal approach to gain easier access and view of the renal vascular hilum approaching the renal artery at the beginning, which reduces bowel mobilization. However, if necessary, the peritoneum was widely opened to achieve complete tumor control. Therefore, the trocar disposition and camera view in the retroperitoneal approach are preferred over the transperitoneal one, although the latter is feasible for kidney masses, especially with low RENAL score.^11^ The same observation seems likely for the open flank approach, as it leads to easy handling of the whole kidney, peritoneum opening, and anterior tumor dissection.

### Outcomes

#### Primary Outcome

To assess surgical outcomes, we applied the Pentafecta score, which consists of the Trifecta score (negative surgical margin, WIT ≤ 25 minutes, no postoperative complications^12^) with additional information on long-term renal function, namely no upstaging to III grade or higher CKD and preservation of eGFR > 90% from baseline to 1 year follow-up.^13^ Renal function was assessed at baseline and 1 year after surgery with estimated glomerular filtration rate (eGFR) according to the modification of diet in renal disease formula. Acute kidney injury was assessed through risk, injury, failure, loss of kidney function and end-stage kidney disease (RIFLE) classification.^14^

#### Secondary Outcomes

The oncological radicality in laparoscopic and open NSS for complex masses was assessed evaluating PSM and follow-up imaging. Moreover, the safety of both approach was tested through the 30-days postoperative complications, which were ranked according to the Clavien–Dindo (CD) classification system,^15^ and considered as minor up to grade 2.

### Statistical Analysis

Categorical variables were reported using absolute frequency and percentage. Continuous variables were assessed for normal distribution with the Kolmogorov–Smirnov test and expressed as mean and SD. Normally distributed variables were compared using independent samples *t*-tests, while the categorical ones with the chi-square test for the independence of measures. Changes in eGFR from baseline were also calculated and analyzed using a Wilcoxon test. All statistical tests were 2-tailed, with *P* < .05 indicating statistical significance. All statistical tests were conducted using SPSS software package version 26 (IBM SPSS Corp., Armonk, NY, USA).

## Results

Two-hundred thirteen patients met the inclusion criteria and were included in the analysis, 76 patients in the LTE group and 137 in the OWR group.

### Baseline Data of Patients

Patients’ demographics and characteristics are shown in Table 1. There were no statistically significant differences in preoperative data between the 2 groups, except for the higher rate of male patients (*P* = .03) and T1b tumors in the LTE group (*P* = .02) and T1a tumors in the OWR group (*P* = .005).

### Intraoperative Outcomes

Table 2 shows intra and perioperative data. Laparoscopic tumor enucleation was performed clampless in all patients, while in only 66 (48.2%) cases in the OWR group. Among the 2 groups, operative time, WIT, 24-h decrease in hemoglobin, PSM, and Trifecta score were similar. Mean estimated blood loss was higher in the OWR group than LTE group (329 ± 269 vs. 249 ± 114 mL, respectively, *P* = .02), as well as mean length of stay (4.7 ± 1.9 vs. 6.1 ± 2.3, *P* < .001), and renorrhaphy rate (137 (100%) vs. 27 (35.5%) cases, *P* < .001).

### Postoperative Outcomes

Minor and major complication rates were comparable between the 2 groups (Table 3). In the LTE group, 14 minor complications occurred (8 CD1 and 6 CD2), whilst 1 patient had an episode of atrial fibrillation and required admission to the intensive care unit (CD 4a), 1 had renal pseudoaneurysm treated with super-selective artery embolization (CD 3a), and 2 had urinary leak from the collecting system that required stent positioning (CD 3b). In the OWR group, 25 patients had minor complications (11 CD1 and 14 CD2), while 2 patients were treated with super-selective artery embolization for bleeding (CD 3b), 2 had urinary leak from the collecting system with stent positioning (CD 3b), and 1 had an acute cardiac ischemic event and required admission to intensive care unit (CD 4a). In the OWR group, only 1 patient with T2 renal tumor required intraoperative conversion to radical nephrectomy because of the insufficient remaining healthy parenchyma.

Mean follow-up was 41 ± 22 months in the LTE group and 45 ± 24 months in the OWR group. No significant difference was reported in terms of preservation of eGFR >90% from baseline and CKD upstaging 1-year after surgery, as shown in Table 4. Finally, the Pentafecta score did not differ between the 2 groups, with 36/77 (46.7%) patients in the LTE group and 62/81 (43.3%) in the OWR group.

## Discussion

Nephron-sparing surgery is preferred over radical nephrectomy for renal function preservation whenever feasible, being associated with a lower cardiovascular risk and mortality rate than radical nephrectomy, particularly in older patients.^16^ Nephron-sparing surgery has shown similar oncological efficacy and safety in long-term outcomes compared to radical nephrectomy with both open^17^ and laparoscopic^18^ techniques. That said, there are currently no clear indications of which approach is best suitable for NSS.^1^ The choice between open and laparoscopic techniques still remains a hot point of debate, and contradictory findings have showed. Rezaeetalab et al^19^ reported similar changes in eGFR after 1 month from surgery and comparable rate of PSM, although higher OT and overall complication rates were reported in patients treated with a laparoscopic approach. Conversely, Abdelhafez et al^20^ showed, in a prospective study involving 356 cases that open NSS was associated with a higher complication rate and longer hospital stay. However, considering only the patients with higher tumor complexity, no significant difference between the 2 techniques occurred. We also found that hospital stay was significantly longer in the OWR group and we argue that this difference was mainly related to the advantage of laparoscopy associated with less pain and less cutting of skin and tissue since postoperative complication were similar between the 2 groups.

A remarkable key for renal function preservation is WIT, particularly in the case of main renal artery clamping, because a significant relationship between prolonged WIT and the development of de novo kidney failure has been demonstrated.^21^ Besides, in a recent critical review, WIT was reported as an independent predictor of renal functional decline, especially in patients aged <60 years.^22^ Our study confirmed that clampless NSS for highly complex renal tumors was always feasible in the LTE group and almost half of the patients in the OWR group. Therefore, the decision to clamp or not should be based on the surgeon’s preference. In the OWR group, mean WIT was low at 7 minutes, with the longest time reaching 20 minutes in only 3 patients. Nevertheless, the mean WIT required in OWR was negligible because the decrease of eGFR 1 year after surgery was low and comparable with the LTE group. Moreover, our findings are in line with the recent CLOCK trial, which demonstrated a similar 6-month e-GFR between off-clamp and on-clamp (14 minutes) partial nephrectomy.^23^ Therefore, a low WIT during NSS might guarantee similar preservation of renal function compared to zero ischemia.

Despite the controversies on the positive association between PSM and cancer survival,^24^ the aim of the complete tumor removal should always be achieved in NSS. In this context, NSS for T2 tumors is a debated topic in the literature as it is a demanding procedure. Shum et al^25^ showed that, despite a significantly higher PSM rate, NSS was associated with a better overall survival than radical nephrectomy. In our study, LTE and OWR groups showed a similar rate of PSM (4% and 5.8%, respectively). Therefore, these results are in line with the reported range of a similar study comparing laparoscopic vs open partial nephrectomy (0%-4% in laparoscopic and 0%-7% in open surgery).^26^ Yet, a recent review demonstrated that NSS was associated with better postoperative renal function preservation, and it was oncologically non-inferior to radical nephrectomy, with a lower likelihood of all-cause mortality (relative risk = 0.78).^27^

Concerning postoperative complications, we showed a low rate of minor complications with no significant difference between the 2 groups. The blood transfusions rate was low at 3.9% and 6.6% in LTE and OWR groups, respectively. Therefore, this study confirms that the clampless approach is not a risk factor for significant bleeding. The major complication rate (CD ≥ 3) was acceptable, with only a 3.9% rate in the laparoscopic group and 3.5% in the open group.

Nephron-sparing surgery is associated with a greater risk of bleeding in lager and highly complex tumors, which can be life threatening in some cases. Nowadays, trans-arterial embolization (TAE) is the preferable option for achieving postoperative hemostasis. Shin et al^28^ reported a higher incidence of postoperative TAE in laparoscopic (5.9%) than in open technique (1.8%). In our study, super-selective TAE was necessary in 1 case in LTE and 2 in the OWR group. The low rate of embolization might be another explanation for the optimal 1-year renal function preservation we achieved in our series.

Postoperative urine leakage is another critical complication after NSS, without evident risk factors other than the surgeon’s lack of experience.^29^ Moreover, regarding complex masses, Stroup et al^30^ reported a positive relationship between the RENAL score and urine leakage. In our series, only 3 patients had a urine leak (1 (1.3%) in LTE and 2 (1.4%) in OWR group) that required only a ureteral stent positioning. Our results demonstrated that a meticulous suturing and adequate hemostasis of renal parenchyma guarantee NSS safety, reducing postoperative blood transfusion, and TAE rate. The Trifecta score was introduced to assess surgical quality, verifying oncological radicality, the technique safety, and limited WIT.

Zargar et al^12^ also included 2 parameters on long-term renal function in addition to Trifecta (Pentafecta) to assess the quality of NSS. In our experience, Pentafecta score was achieved in a similar number of patients in groups. Therefore, our results confirm that keeping WIT low did not significantly affect renal parenchyma, allowing a good preservation of kidney function 1 year after surgery similarly to a clamp less approach.

Our study has some limitations starting with its retrospective nature. Nevertheless, the study depicts a real-life setting and may reflect the results in high-volume referral centers. Second, almost half of the patients had clamped their arteries in open NSS. However, the decrease in eGFR did not significantly differ between baseline and 1-year follow-up, confirming that a low WIT is safe for renal function preservation. Finally, all procedures were performed by 2 experienced surgeons, and less skilled surgeons could achieve different outcomes.

In conclusion, the open NSS open technique remains a viable option in NSS, having comparable LOS and postoperative complications compared to laparoscopy, despite higher blood loss and longer postoperative stay. Both laparoscopic and open NSS offered satisfactory functional and oncological outcomes for renal tumors with a RENAL nephrometic score ≥7.

## Figures and Tables

**Table 1.** Descriptive Characteristics of Patients in the LTE and OWR Groups and *P-*Value

Variables	LTE Group (n = 76)	OWR Group (n = 137)	*P*
Age, years	64 ± 13	64 ± 12	.73
BMI, kg/m^2^	26.9 ± 3.9	26.1 ± 2.4	.35
Sex			.03
Male	48 (63.2)	65 (47.4)	
Female	28 (36.8)	72 (52.6)	
Charlson comorbidity index			.1
2-4	31 (40.8)	72 (52.5)	
5 or higher	45 (59.2)	65 (41.5)	
ASA score			
Grade 1	9 (11.8)	28 (20.4)	.11
Grade 2	50 (65.8)	90 (65.7)	.99
Grade 3	17 (22.4)	19 (13.9)	.06
Tumor side			.98
Right	37 (48.9)	67 (48.9)	
Left	39 (51.1)	70 (51.1)	
Tumor largest dimension, mm	47 ± 15	47 ± 21	.92
T stage			
T1a	17 (22.4)	58 (42.3)	.005
T1b	43 (56.6)	55 (40.2)	.02
T2	16 (21.1)	24 (17.5)	.53
RENAL score			.13
Intermediate (7-9)	69 (90.8)	114 (83.2)	
High (10-12)	7 (9.2)	23 (18.2)	
Preoperative eGFR, mL/min	94.1 ± 34.6	94.5 ± 33.6	.94
Number of patients with chronic renal failure			
Grade II	22 (29)	44 (32.1)	.63
Grade III	11 (14.5)	17 (12.4)	.67
Grade IV	1 (1.3)	2 (1.5)	.93
Grade V	1 (1.3)	1 (0.7)	.67

Data are presented as means (SD) and frequencies (proportions).

ASA, American Society of Anaesthesiologists; BMI, body mass index; eGFR, estimated glomerular filtration rate; LTE, laparoscopic tumor enucleation; OWR, open wedge resection; RENAL, radius exophytic/endophytic nearness anterior/posterior location.

**Table 2.** Perioperative Data of Patients Related to the LTE and OWR Groups and *P*-value

Variables	LTE Group (n = 76)	OWR Group (n = 137)	*P*
Operative time, min	132 ± 49	144 ± 52	.11
Estimated blood loss, mL	249 ± 114	329 ± 269	.02
WIT, minutes	0	7.5 (0-14)^*^	.7
WIT			
No ischemia	76 (100)	66 (48.2)	
*T* < 25 minutes	0 (0)	71 (51.8)	
*T* ≥ 25 minutes	0 (0)	3 (2.2)	
Conversion to radical nephrectomy			.23
Yes	2 (2.6)	1 (0.7)	
No	74 (97.4)	136 (99.3)	
Renorrhaphy			<.001
Yes	27 (35.5)	137 (100)	
No	49 (64.5)	0 (0)	
24-Hour decrease in hemoglobin, g/dL	1.9 ± 1.6	1.8 ± 1.4	.41
Length of stay, days	4.7 ± 1.9	6.1 ± 2.3	<.001
At least 1 postoperative complication			.97
Yes	17 (22.1)	31 (22.6)	
No	45 (77.9)	106 (77.4)	
CD classification			
CD1	8 (10.5)	12 (8.8)	.67
CD2	6 (7.8)	14 (10.2)	.58
CD3a or more	3 (3.9)	5 (3.7)	.91
Positive surgical margin			.55
Yes	3 (4)	8 (5.8)	
No	73 (96)	129 (95.2)	
Trifecta score			.78
Yes	59 (77.6)	104 (75.9)	
No	17 (22.4)	33 (24.1)	
1YPO eGFR, mL/min	88.2 ± 30	87.4 ± 32.8	.87

Data are presented as means (SD) and frequencies (proportions).

^*^Variables are not normally distributed, which were reported with medians (interquartile ranges).

1YPO, 1-year from surgery; CD, Clavien–Dindo; eGFR, estimated glomerular filtration rate; LTE, laparoscopic tumor enucleation; OWR, open wedge resection; WIT, warm ischemia time.

**Table 3.** Postoperative Complications of Patients Related to the LTE and OWR Groups and *P*-Value

Complication	Treatment	LTE Group (n = 76)	OWR Group (n = 137)	*P*
Clavien–Dindo 1		8 (10.5)	11 (8.8)	.67
Fever	Antipyretics	4 (5.2)	7 (5.1)	
Pleural injury	No further treatment	2 (2.6)	1 (0.7)	
Postoperative anemia	No further treatment	2 (2.6)	3 (2.2)	
Clavien–Dindo 2		6 (7.8)	14 (10.2)	.58
Pneumonia	Antibiotics	1 (1.3)	2 (1.5)	
Postoperative hemorrhage	Blood transfusion	3 (3.9)	9 (6.6)	
Acute kidney injury	Diuretics administration	2 (2.6)	3 (2.2)	
Clavien–Dindo 3		2 (2.6)	4 (2.9)	.9
Renal pseudoaneurysm	Super-selective embolization	1 (1.3)	2 (1.4)	
Urinary leak	Stent insertion	1 (1.3)	2 (1.4)	
Clavien–Dindo 4		1 (1.3)	1 (0.7)	.67
Atrial fibrillation	Admission to ICU	1 (1.3)	0 (0)	
Myocardial infarction	Admission to ICU	0 (0)	1 (0.7)	

Data are presented as frequencies (proportions).

ICU, intensive care unit; LTE, laparoscopic tumor enucleation; OWR, open wedge resection.

**Table 4.** Pentafecta Outcomes of Patients Related to the LTE and OWR Groups and *P*-Value

Complication	LTE Group (n = 76)	OWR Group (n = 137)	*P*
WIT ≥ 25 minutes			.19
Yes	0 (0)	3 (2.1)	
No	76 (100)	134 (97.9)	
At least 1 postoperative complication			.97
Yes	17 (22.1)	31 (22.7)	
No	45 (77.9)	106 (77.3)	
Positive surgical margin			.55
Yes	3 (4)	8 (5.6)	
No	73 (96)	129 (95.4)	
Preservation of eGFR >90% at 1YPO			.82
Yes	50 (65.8)	88 (64.2)	
No	26 (34.2)	49 (35.8)	
CKD upstaging at 1YPO			.7
Yes	5 (7)	11 (8)	
No	71 (93)	126 (92)	
Pentafecta score			.83
Yes	35 (46.1)	61 (44.5)	
No	41 (53.9)	76 (55.5)	

1YPO, 1 year from surgery; CKD, chronic kidney disease; eGFR, estimated glomerular filtration; LTE, laparoscopic tumor enucleation; OWR, open wedge resection; WIT, warm ischemia time.

Data are presented as frequencies (proportions).
